# Inverse Solvent Isotope Effects in Enzyme-Catalyzed Reactions

**DOI:** 10.3390/molecules25081933

**Published:** 2020-04-21

**Authors:** Patrick L. Fernandez, Andrew S. Murkin

**Affiliations:** Department of Chemistry, University at Buffalo, The State University of New York, Buffalo, NY 14260-3000, USA; plfernan@buffalo.edu

**Keywords:** solvent isotope effects, inverse isotope effects, equilibrium isotope effects, kinetic isotope effects, fractionation factor

## Abstract

Solvent isotope effects have long been used as a mechanistic tool for determining enzyme mechanisms. Most commonly, macroscopic rate constants such as *k*_cat_ and *k*_cat_/*K*_m_ are found to decrease when the reaction is performed in D_2_O for a variety of reasons including the transfer of protons. Under certain circumstances, these constants are found to increase, in what is termed an inverse solvent kinetic isotope effect (SKIE), which can be a diagnostic mechanistic feature. Generally, these phenomena can be attributed to an inverse solvent equilibrium isotope effect on a rapid equilibrium preceding the rate-limiting step(s). This review surveys inverse SKIEs in enzyme-catalyzed reactions by assessing their underlying origins in common mechanistic themes. Case studies for each category are presented, and the mechanistic implications are put into context. It is hoped that readers may find the illustrative examples valuable in planning and interpreting solvent isotope effect experiments.

## 1. Introduction

Solvent isotope effects (SIEs) can be a powerful tool for studying the mechanisms of enzyme-catalyzed reactions. SIEs report on solvent-sensitive steps in a mechanism—those steps that are involved or implicated in bond cleavage and bond formation of solvent-exchangeable hydrons as well as changes in the hydrogen bond network. There exist resources and literature on many aspects of this topic, most notably from the early reviews by Schowen and Schowen [1] and more recently by Quinn [2].

This review focuses on inverse SIEs—that is, SIEs in which the reactions are favored in D_2_O rather than H_2_O—and offers several origins to aid in their mechanistic interpretation. The much more common normal SIEs (those favoring H_2_O) have been discussed in great detail in the above reviews and elsewhere, but inverse SIEs have generally only received limited attention. As will be explained in the following section, unlike normal SIEs, which can be the result of solvent kinetic isotope effects (SKIEs), solvent equilibrium isotope effects (SEIEs), or a combination of both, almost all inverse SIEs are dominated by a SEIE (i.e., an equilibrium that is more favorable in D_2_O). As such, the observation of an inverse SIE can be an especially diagnostic feature.

The remaining sections highlight examples of SIEs that fall into major themes: (1) cysteine thiols, (2) metal-bound water, (3) low-barrier hydrogen bonds (LBHBs), (4) medium effects, and (5) conformational changes. While this review is not intended to be comprehensive of all works on inverse SIEs, it presents a selection of examples spanning from early to recent investigations.

## 2. Theory of Solvent Isotope Effects

**Figure 1:**
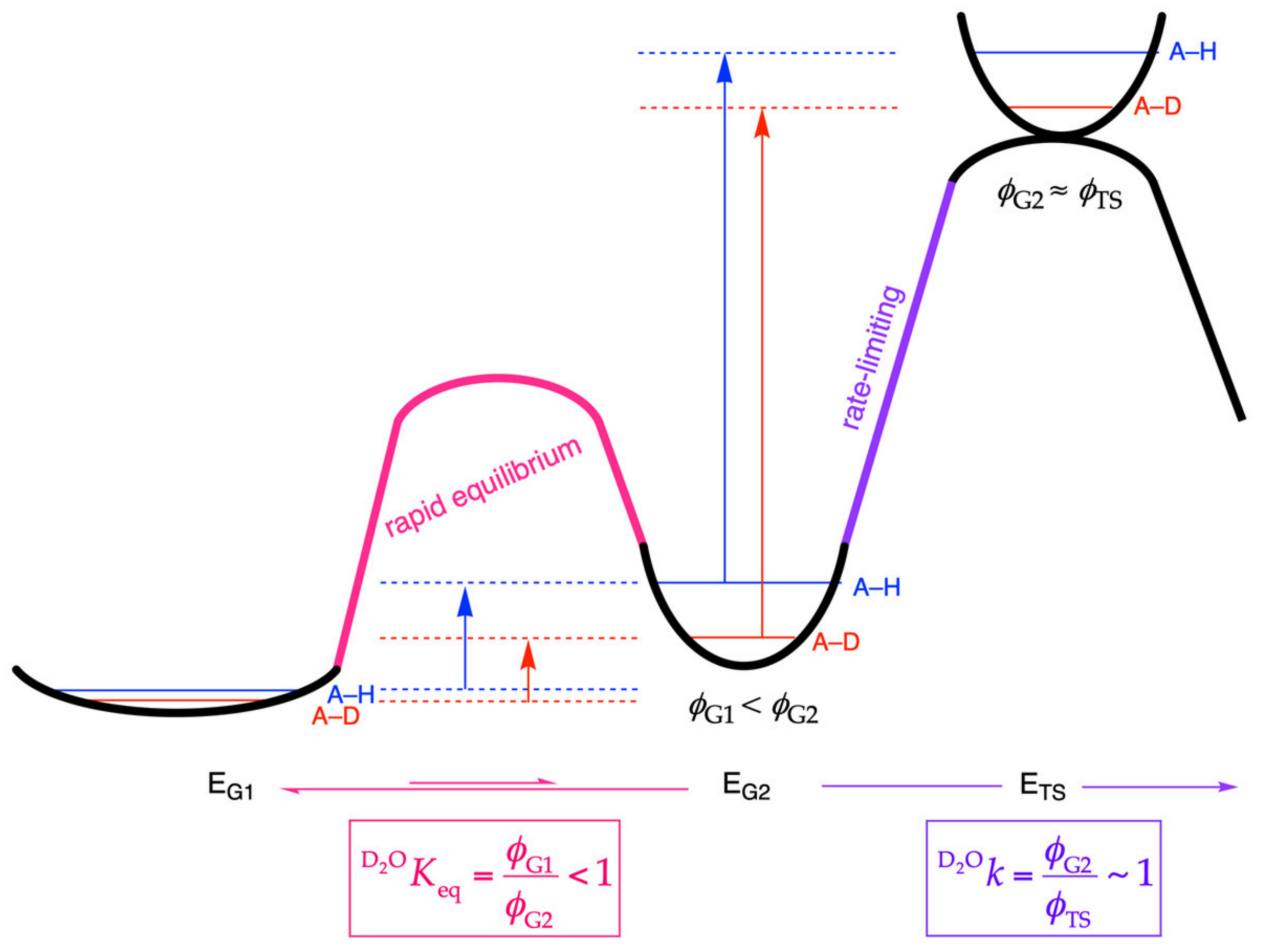
Reaction coordinate diagram of a solvent-sensitive rapid equilibrium preceding a rate-limiting step that results in inverse solvent isotope effects. Horizontal lines represent zero-point energy levels, reflecting the mass of the hydron and the stiffness of the bond in which it is involved. When the stiffness of the bond increases, as shown in the rapid-equilibrium step (pink), the zero-point energy difference between the two isotopes increases relative to the reactant ground state (G1), leading to accumulation of deuterium in the intermediate ground state (G2); this is reflected in an increase in the fractionation factor (*ϕ*; Equation (1)) for this step and an inverse solvent equilibrium isotope effect (SEIE) (i.e., ^D2O^*K*_eq_ < 1). If the stiffness of the solvent-exchangeable hydron is not altered in the transition state (TS) of a subsequent rate-limiting step (purple), then no SKIE will be contributed for this step (i.e., ^D2O^*k* ~ 1). The observed inverse solvent kinetic isotope effect (SKIE) (^D2O^*k*_obs_) for the overall process will be inverse (Equation (2)). A = solvent-exchangeable group; E = enzyme.

The transfer of an exchangeable proton in a rate-limiting step frequently results in an observed SKIE. In contrast to secondary deuterium isotope effects, which reflect vibrational changes orthogonal to the reaction coordinate and can have inverse values (e.g., hybridization changes from *sp*^2^ to *sp*^3^), the transfer of solvent-sensitive protons yields primary deuterium isotope effects that occur along the reaction coordinate (Figure 1). During this event, the bond to the donor weakens in the transition state, yielding a *normal* SKIE (i.e., ^D2O^*k* ≡ *k*_H2O_/*k*_D2O_ > 1). Thus, except for those involved in LBHBs (discussed in Section 5), protons in flight in a rate-limiting step necessarily produce normal SIE contributions (Figure 1, purple segment). It may therefore seem counterintuitive that inverse SKIEs are observed. Although inverse SKIEs are useful in ruling out solvent-sensitive steps as being rate limiting, they are also informative in revealing the nature of other steps in the mechanism, as will be discussed in the later sections.

All SIEs can be analyzed in terms of fractionation factors. Fractionation factors, *ϕ*, describe the preference of an exchangeable group (A–H or A–D) for D over H, relative to the isotope ratio in the bulk solvent (Equation (1)).

(1) $$\phi_{A}=\frac{\left[A–D\right]{[H}_{2}O]}{\left[A–H\right]{[D}_{2}O]}$$

The SIE of any reaction is the ratio of the fractionation factors at the initial state to the fractionation factors at the final state. For an intrinsic SKIE (that is, a SKIE on a single microscopic rate constant), this is expressed as ^D2O^*k* = *ϕ*_R_/*ϕ*_TS_; for a SEIE, this is expressed as ^D2O^*K*_eq_ = *ϕ*_R_/*ϕ*_P_. Differing from intrinsic SKIEs, SEIEs influence the relative isotopic populations between two ground states in equilibrium (Figure 1, pink segment); when the reactant fractionation factor is smaller than that of the product, the SEIE will be inverse. Common fractionation factors relevant to enzyme-catalyzed reactions are summarized in Table 1. Note that throughout this document, the widely used symbol L (from lyonium or lyate) is used to denote either H or D, depending on the context.

When the SEIE (1) precedes a rate-limiting step and (2) occurs in a rapid equilibrium (Figure 1), it manifests in the observed SKIE. For a simple two-step process, this can be expressed as the product of the two isotope effects (Equation (2)).

(2) kobsD2O=KeqD2OkD2O

This relationship shows that an inverse ^D2O^*K*_eq_ can diminish or even overturn a normal ^D2O^*k* if it is large enough, resulting in an overall inverse observed SKIE. The rapid-equilibrium assumption is necessary to avoid expression of any normal SKIE that would otherwise accompany that step, which could mask the inverse SIE. In many situations the influence of the SEIE on the observed SKIE depends on the magnitude of *K*_eq_, ranging from unity for a highly favorable equilibrium to full expression of the inverse SEIE for a highly unfavorable equilibrium [9,10,11]. For this reason, the solvent-sensitive equilibria in this review are presented as favoring reactants, though this is not a strict requirement, as the true expression of the SEIE is mechanism dependent.

### The Importance of pH Independence

There are two important considerations related to pL when performing SKIE experiments. First, when using a glass electrode that has been calibrated with buffers prepared in H_2_O to measure the pD of solutions in D_2_O, one must correct the measured pH meter reading according to Equation (3) [12].

pD = pH meter reading + 0.4(3)

**Figure 2:**
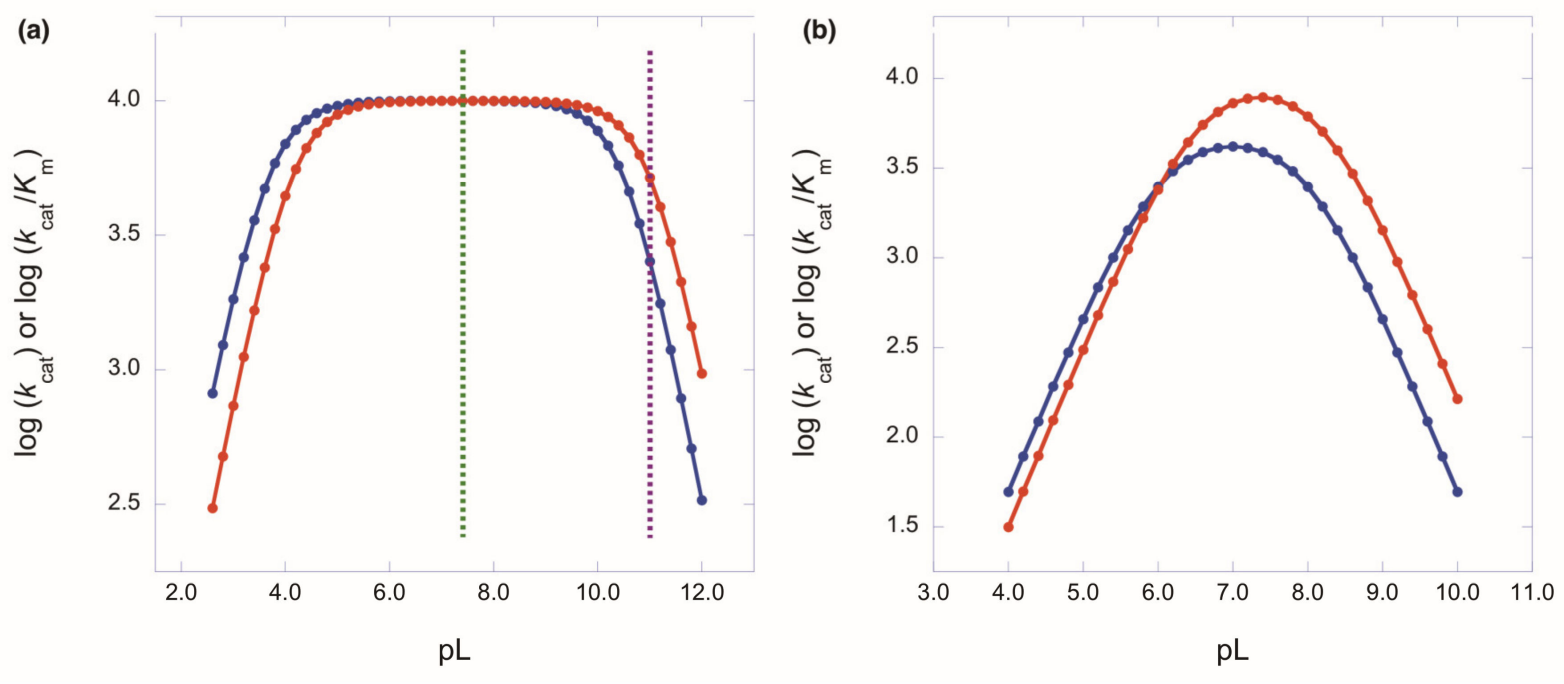
Simulated pL–rate profiles in H_2_O (blue) and in D_2_O (red). (**a**) SKIE = 1; p*K*_a1_ = 3.65 (RCO_2_L); p*K*_a2_ = 10.53 (RNL_3_^+^). The green dashed line represents a suitable pL for measuring SKIEs accurately, while the purple line represents a pL that would lead to the false measurement of an inverse SKIE. (**b**) SKIE = 0.5; p*K*_a1_ = 6.0 (RSL); p*K*_a2_ = 8.0 (RNL_3_^+^). A plateau is absent because the two p*K*_a_ values do not differ enough.

Second, solvent isotope effects must be determined at equivalent positions in the respective pL–rate profiles, preferably in a pL-independent region (e.g., Figure 2a, green line). The reason for this requirement is that the p*K*_a_ of most monoprotic Brønsted acids increases by ~0.5 in D_2_O relative to H_2_O (Figure 2).

The origin of this effect is the inverse fractionation factor of the lyonium ion (*ϕ*_L3O+_ = 0.69), according to Equation (4),

(4) $${pK}_{D_{2}O}={\;pK}_{H_{2}O}+\log\left(\phi_{AL}/\phi_{L_{3}O^{+}}^{3}\right)\approx {pK}_{H_{2}O}+0.48$$

where *ϕ*_AL_ ~ 1 for most acids. Note that if a fixed pL were chosen in the basic limb (Figure 2a, purple line), the *calculated* SKIE would be incorrectly judged as inverse, despite the absence of an actual SKIE. For profiles that do not have plateaus because the p*K*_a_ values of the ascending and descending limbs are close (Figure 2b), the pL-independent parameters, C*, can be obtained by fitting to Equation (5) (or its non-logarithmic form) [13].

(5) $$\mathrm{logC}=\log\left[\frac{C^{*}}{1+10^{-\left(pL\;-{pK}_{a1}\right)}+10^{-\left({pK}_{a2}-pL\right)}}\right]$$

## 3. Cysteine Thiols

**Scheme 1:**
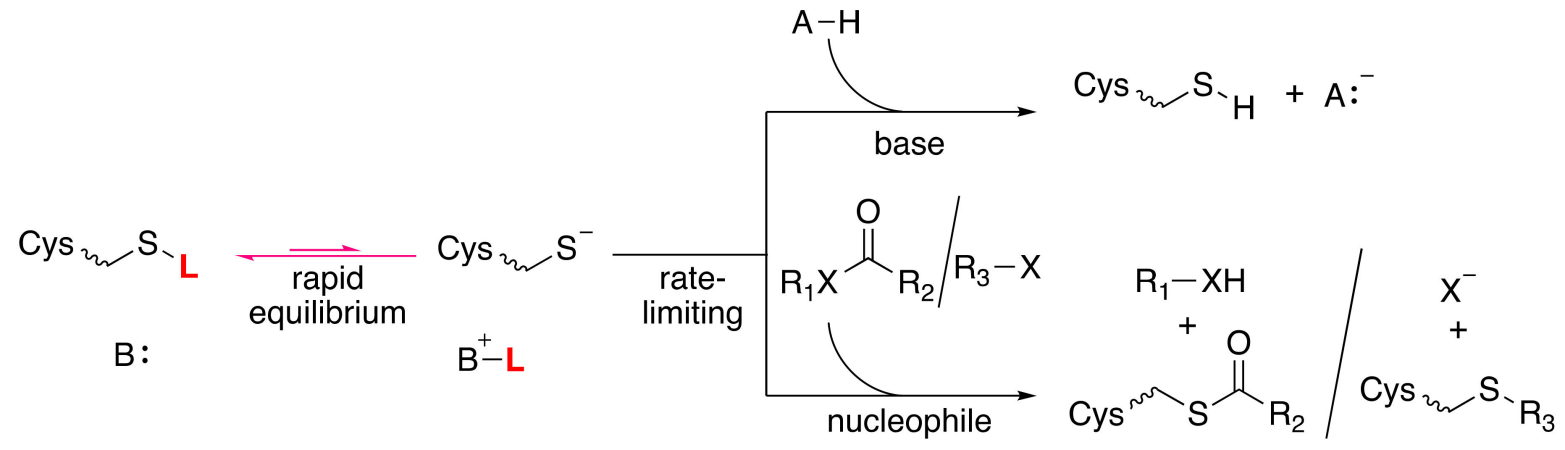
Examples of thiolate utility in enzyme-catalyzed reactions. A–H = Brønsted acid (depicted as not solvent exchangeable; a solvent-exchangeable acid would contribute a normal SIE that would mask the preceding inverse SEIE); B = Brønsted base; X = O, NR, halide.

Thiols have exceptionally large inverse fractionation factors: *ϕ*_RSL_ = 0.40–0.46 (nonenzymatic thiols) [1] and *ϕ*_RSL_ = 0.55 (catalytic cysteine in proline racemase) [5]. Many enzymes employ cysteine thiolates as nucleophiles (e.g., acylation and alkylation) or Brønsted bases. If the predominant form of this residue is the thiol but it exists in rapid equilibrium with the reactive thiolate, this equilibrium will be subject to a SEIE (Scheme 1). Stated differently, the thiolate form of the free enzyme is enriched in D_2_O by a factor of approximately 1/*ϕ*_RSL_ ≈ 2. Some examples are summarized in Table 2.

### 3.1. Cysteine Proteases: Papain and Cathepsin C

Cysteine proteases catalyze peptide hydrolysis via an acyl-enzyme intermediate formed by nucleophilic attack by a cysteine–histidine dyad. The solvent isotope effects on *k*_cat_/*K*_m_ for the acylation half-reaction of the hydrolysis of *N*-protected amino acid amides by papain were found to be inverse (0.46–0.63) [14]. This is attributed to the prototropic tautomerism exhibited by the catalytic cysteine–histidine dyad that generates the reactive thiolate species (Scheme 1). When papain was alkylated by bromoacetamide, the SKIE was also inverse (*k*_alk_ = 0.74), implicating the same reactive thiolate for this activity [14]. These magnitudes agree with those measured for non-enzymatic models of thiolate alkylation by α-haloacetic acid derivatives using cysteine (0.53–0.85) [15]. These inverse effects agree with the SEIE on thiol deprotonation by an amine (*ϕ*_RSL_ = 0.43) [19].

A similarly inverse ^D2O^(*k*_cat_/*K*_m_) of 0.71 ± 0.14 was observed for cathepsin C-catalyzed hydrolysis of Ser-Tyr-AMC, a peptide with a fluorogenic leaving group [9]. Like other cysteine proteases, cathepsin C utilizes the same catalytic dyad rapid equilibrium prior to substrate binding. Because there is proton transfer in the downstream partially rate-limiting formation and/or collapse of the tetrahedral intermediate, the above SKIE on *k*_cat_/*K*_m_ reflects a tautomeric SEIE of 0.39 that is masked by a normal SKIE on acylation (^D^*k*_ac_ = 1.31 ± 0.04) [9]. In other cysteine proteases, the normal ^D^*k*_ac_ may well be large enough to completely nullify the inverse SEIE. For example, ^D2O^(*k*_cat_/*K*_m_) = 1.14–1.4 for cruzain [20]. However, it may also be the case that the preequilibrium is “silent” because the thiol form is not as strongly favored.

### 3.2. Protein Arginine Deiminase

Protein arginine deiminase (PAD) is one of several isozymes that catalyze the hydrolysis of arginine residues on protein substrates including histones and fibrinogen. Catalysis is achieved by nucleophilic attack by a cysteine residue on the guanidinium group in concert with proton transfer from a histidine residue [16]. Inverse SIEs were measured for the isozymes PAD2 and PAD4: ^D2O^(*k*_cat_/*K*_m_)_PAD2_ = 0.8 ± 0.1 [16] and ^D2O^(*k*_cat_/*K*_m_)_PAD4_ = 0.43 ± 0.07 [17]. The larger inverse SIE for PAD4 was interpreted as the result of a reverse protonation mechanism in which the predominant thiol-imidazole pair must shift to the thiolate–imidazolium form for catalysis [17], while PAD2 is believed to utilize a substrate-assisted mechanism in which the substrate’s guanidinium group lowers the p*K*_a_ of the reactive Cys647 [16]. Note that because ^D2O^(*k*_cat_/*K*_m_)_PAD4_ is close to the lower theoretical limit for the thiol-thiolate SEIE, subsequent mechanistic steps involving transfer of a solvent-derived hydron are not significantly rate limiting on *k*_cat_/*K*_m_, a conclusion that is consistent with the relatively small ^D^*k*_cat_ of 1.25 [17].

### 3.3. Isocitrate Lyase

Isocitrate lyase catalyzes the reversible aldol addition of succinate to glyoxylate. In this reaction direction, it employs the thiolate of Cys191 as a Brønsted base to abstract an α-proton from succinate. A SKIE on *k*_cat_/*K*_m_ of 0.56 ± 0.07 was measured, while the corresponding SKIE on *k*_cat_ was normal (^D2O^*k*_cat_ = 1.7 ± 0.4) [10]. The inverse SKIE was attributed to an SEIE arising from the rapid equilibrium of thiolate formation prior to binding of succinate, while the normal SKIE was attributed to the downstream transfer of a solvent-derived hydron, presumably involved in protonation of the carbonyl oxygen of glyoxylate. Interestingly, an inverse SKIE of 0.40 ± 0.03 was also measured on *k*_inact_/*K*_I_ for 3-nitropropionate [10], a succinate analogue that reacts covalently with Cys191, a result that can be attributed to the same pre-binding equilibrium and a requirement for the minor thiolate form [21].

## 4. Metal-bound Water

**Scheme 2:**
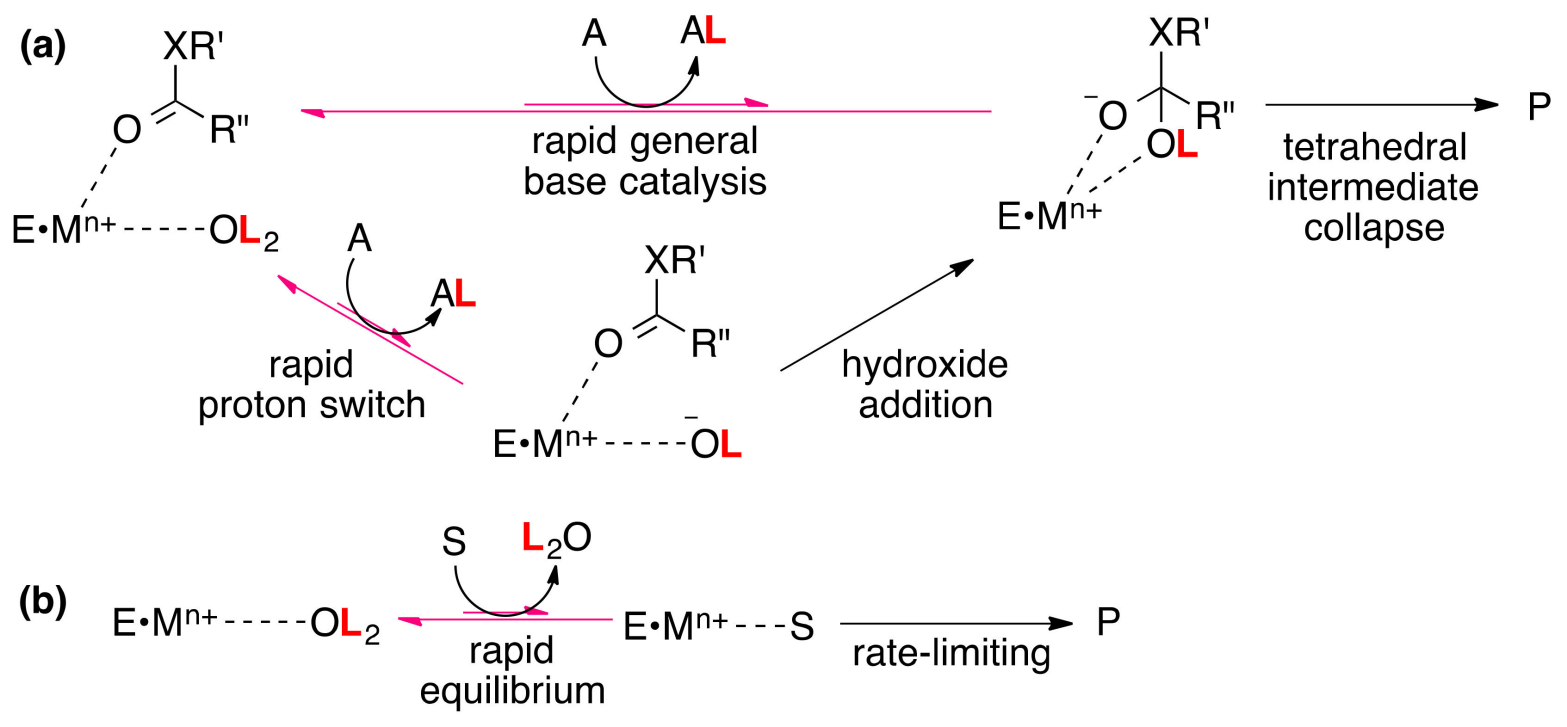
Common themes for metal-bound water implicated in inverse solvent isotope effects. (**a**) Nucleophilic addition of metal-bound hydroxide generated from metal-activated water. This process is accomplished either by general base catalysis in a rapid equilibrium or by rapid formation of hydroxide followed by nucleophilic attack. X = O, NR. (**b**) Dissociation of water by substrate. If the displacement occurs as the substrate binds to the enzyme, a SKIE would only be reflected in *k*_cat_/*K*_m_; if the substrate was already bound prior to displacement, a SKIE may be observed on *k*_cat_.

Due to the partial positive charge on oxygen when water is coordinated to a metal, the fractionation factors of metal-bound water are inverse, reminiscent of hydrons on the lyonium ion (*ϕ*_L3O+_ = 0.69). Reactions that first proceed through rapid-equilibrium changes to metal-bound water prior to a rate-limiting step can give rise to inverse solvent isotope effects. There are two common processes implicating metal-bound water in this way: (1) displacement/dissociation of water due to substrate binding and (2) addition of water/hydroxide to a group to be hydrated or hydrolyzed (Scheme 2). Examples are summarized in Table 3.

### 4.1. Carbonic Anhydrase

Carbonic anhydrase, which primarily catalyzes the interconversion of carbon dioxide and bicarbonate using an active-site Zn^2+^, also catalyzes ester hydrolysis. The esterase activity of human carbonic anhydrase C on *p*-nitrophenyl acetate (pNPA) was reported to give an inverse SIE on the apparent second-order rate constant, ^D2O^(*k*_cat_/*K*_m_) = 0.73 [22]. This was attributed to a Zn^2+^-bound hydroxide in rapid-equilibrium formation of a tetrahedral intermediate that slowly collapses to give the carboxylate. This value is in good agreement with the fractionation factor of Co^2+^-bound hydroxide in carbonic anhydrase (*ϕ*_Co-LOL_ = 0.74) [8], which suggests that the fractionation factor of hydrons at the tetrahedral intermediate is close to unity.

### 4.2. Metal-Dependent Amidohydrolases: Thermolysin, Stromelysin, and Desuccinylase

Like carbonic anhydrase, metalloproteinases utilize a metal ion as a Lewis acid to lower the p*K*_a_ of water, activating it as a nucleophile. Thermolysin-catalyzed hydrolysis of *N*-(3-(2-furyl)acryloyl)-Gly-Leu-NH_2_ presented an inverse solvent isotope effect on *k*_cat_/*K*_m_ of 0.80 ± 0.02 [23]. Hydrolysis of another seven similar substrates yielded comparable results: ^D2O^(*k*_cat_/*K*_m_) ≈ 0.74 [24]. These inverse effects are notable in that they rule out general base catalysis in the step(s) that limit *k*_cat_/*K*_m_, as such a process would make a substantial normal contribution to the KIE. Instead, the authors proposed two alternative pathways to explain the origin of the inverse SKIE (Scheme 2a): (1) general base catalysis from Glu143 occurs in a rapid equilibrium followed by rate-limiting collapse of a zwitterionic tetrahedral intermediate, or (2) hydroxide is first generated by rapid-equilibrium hydron transfer to Glu143 (a so-called “proton switch”) followed by partially rate-limiting nucleophilic attack. Inverse SEIE may arise from these equilibria.

Stein and coworkers performed a similar analysis on stromelysin and again measured an inverse SIE on *k*_cat_/*K*_m_: ^D2O^(*k*_cat_/*K*_m_) = 0.80 ± 0.02 [25]. This SKIE is similarly consistent with a rapid-equilibrium reaction of a Zn^2+^-bound water, whether by general base catalysis or via hydroxide, from the unbound enzyme with substrate to generate a tetrahedral intermediate with a fractionation factor of unity. Interestingly, the SKIE on *k*_cat_ was normal (^D^*k*_cat_ = 1.58 ± 0.05), indicating that the two SKIEs reflect different rate-limiting steps. The authors suggested that collapse of the tetrahedral intermediate represents the first irreversible step (and thus the final step reflected in *k*_cat_/*K*_m_) and that the origin of the normal ^D^*k*_cat_ is the displacement of product by water, a process that is proposed to involve proton catalytic bridges.

Succinyl-diaminopimelate desuccinylase utilizes a Zn^2+^- or Co^2+^-bound water to hydrolyze *N*-succinyl-l,l-diaminopimelate to liberate l,l-diaminopimelate, which is a building block of the peptidoglycan layer. The SKIEs for both macroscopic rate constants are similar [^D2O^(*k*_cat_/*K*_m_) = 0.62 ± 0.06 and ^D2O^*k*_cat_ = 0.78 ± 0.05], indicating that both parameters share a common solvent-sensitive step [26]. This step has been proposed to be acylation, and the SKIEs were attributed to the inverse fractionation factor of the metal-bound hydroxide and to the unity fractionation factor assumed for the tetrahedral intermediate.

### 4.3. Alcohol Dehydrogenase

**Scheme 3:**
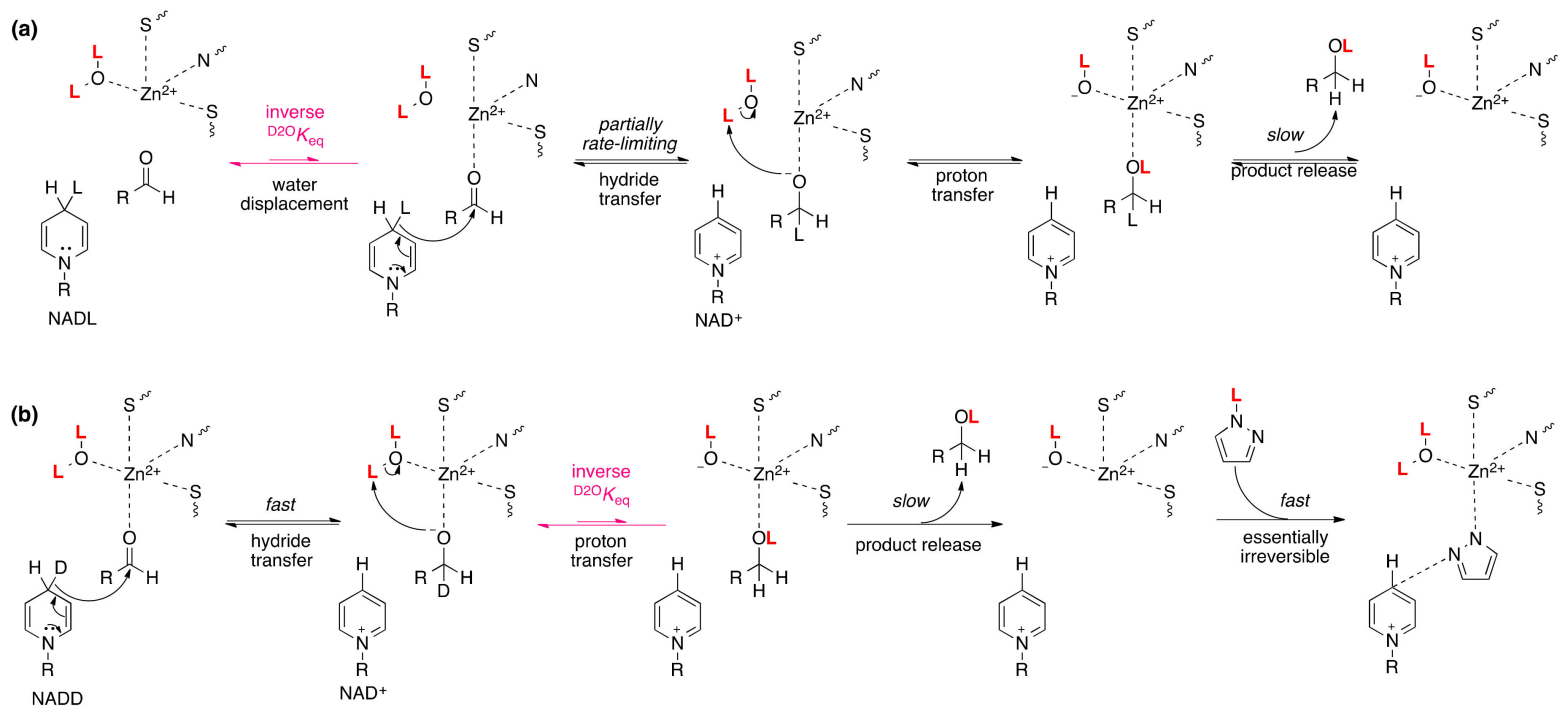
Proposed mechanisms for alcohol dehydrogenases featuring inverse solvent isotope effects associated with rapid equilibria. (**a**) Ligand displacement of a Zn^2+^-bound water by the substrate gives rise to an inverse SEIE in the yeast alcohol dehydrogenase (YADH)-catalyzed reduction of *p*-methoxybenzaldehyde. (**b**) Transfer of a hydron from a Zn^2+^-bound water to the alkoxide intermediate yields an inverse SEIE in the liver alcohol dehydrogenase (LADH)-catalyzed reduction of 4-(2′-imidazolyl)azobenzaldehyde by NADD.

Alcohol dehydrogenases are a class of Zn^2+^-dependent oxidoreductases that catalyze the reversible oxidation of primary and secondary alcohols to aldehydes and ketones, respectively, via transfer of a hydride to NAD^+^ and release of a proton. Solvent isotope effects have been measured by steady-state kinetics for yeast alcohol dehydrogenase (YADH) and by transient kinetics for liver alcohol dehydrogenase (LADH). While ^D2O^*k*_cat_ for YADH was found to be only 1.20 ± 0.09 for the oxidation of *p*-methoxybenzyl alcohol, ^D2O^*k*_cat_ in the reverse direction using *p*-methoxybenzaldehyde was 0.50 ± 0.05 with NADH and 0.58 ± 0.06 with NADD [27]. The small SKIE in the oxidation direction rules out concerted hydride transfer and alcohol deprotonation. In the reduction direction, ^D2O^*k*_cat_ was unaffected by the isotopic nature of the hydride donor, which itself is subject to a primary deuterium KIE on *k*_cat_ of 3.4 [32], indicating that the steps giving rise to the two KIEs are uncoupled. Klinman and coworkers suggested that the inverse SKIE is due to displacement of Zn^2+^-bound water (*ϕ*_Zn–LOL_ = 0.35–0.54) in the primary coordination sphere by the neighboring bound substrate (aldehyde) prior to the chemical reaction (Scheme 3a).

Schmidt et al. measured single-turnover kinetics for the LADH-catalyzed reduction of 4-(2′-imidazolyl)azobenzaldehyde and observed two transients [28]. The fast phase, which corresponds to the hydride-transfer step, was insensitive to solvent (^D2O^*k*_fast_ = 1.0 ± 0.1), while the slow phase was subject to an inverse SKIE (^D2O^*k*_slow_ = 0.394 ± 0.005) at pL 8.75 across several subsaturating concentrations. The slow phase was interpreted as representing the rate-limiting release of the product alcohol. Differing from YADH, however, Schmidt et al. suggested that the aldehyde does not displace Zn^2+^-bound water but instead occupies a fifth coordination site (Scheme 3b). As the origin of the inverse SKIE, the authors favored the rapid-equilibrium transfer of a proton from Zn^2+^-bound water, which has an inverse fractionation factor, to the alkoxide prior to rate-limiting alcohol release; the fractionation factors of the Zn^2+^-bound hydroxide and alcohol product are assumed to be unity [28].

### 4.4. Malate Synthase

Malate synthase catalyzes the Claisen-like condensation of glyoxylate with acetyl-CoA to yield malate. The reaction proceeds by deprotonation of the acetyl group, which attacks glyoxylate’s carbonyl, followed by hydrolysis of the thioester by a Mg^2+^-bound hydroxide ion. A primary deuterium KIE on *k*_cat_/*K*_m_ was measured for (*methyl*-^2^H_3_)acetyl-CoA [^D^(*k*_cat_/*K*_AcCoA_) = 2.3 ± 0.3] using malate synthase from *Mycobacterium tuberculosis*, indicating that deprotonation is rate limiting [29]. Interestingly, while ^D2O^(*k*_cat_/*K*_AcCoA_) was unity with unlabeled substrate, an inverse SKIE of 0.6 ± 0.1 was measured using [*methyl*-^2^H_3_]acetyl-CoA. This result implicates a solvent-sensitive equilibrium prior to deprotonation of acetyl-CoA that becomes exposed when the barrier to the subsequent rate-limiting deprotonation is elevated. While the authors were unable to conclusively determine the origin of the inverse SKIE, they suggested the involvement of the water molecules coordinated to the catalytic Mg^2+^ ion. We speculate that the nucleophilic water loses a hydron to a nearby base, similar to the proton switch in Scheme 2a, prior to deprotonation of acetyl-CoA.

### 4.5. Hypoxia-Inducible Factor-α (HIF) Hydroxylases: Prolyl Hydroxylase 2 (PDH2) and Factor-Inhibiting HIF (FIH)

Prolyl hydroxylase 2 (PDH2) and factor-inhibiting HIF (FIH) are two enzymes that use non-heme Fe^2+^ and molecular oxygen to hydroxylate specific residues in the hypoxia-inducible factor-α (HIF), a transcription factor that serves an important role in the cellular response to oxygen levels. An inverse SIE on the apparent (subsaturating [O_2_]) *k*_cat_ was measured for the acidic and basic forms of PDH2: ^D2O^*k*_cat_ = 0.91 ± 0.03 (acidic) and 0.9 ± 0.1 (basic). This was attributed to a SEIE resulting from the displacement of Fe^2+^-bound water after peptide binding but prior to O_2_ binding [11]. Assuming a fractionation factor of 0.7 for each hydron in water bound to Fe^2+^, the SEIE was estimated to be ~0.49. The degree to which this SEIE is expressed in ^D2O^*k*_cat_ depends in part on the equilibrium constant for water dissociation, with maximum expression when water binding is strongly favored (i.e., *K*_eq_ << 1) and complete suppression when water release is strongly favored (*K*_eq_ >> 1). Because ^D2O^*k*_cat_ < 1, the authors concluded that *K*_eq_ < 1, although this also depends on the relative magnitudes of the *kinetic* SIE for this step as well as the commitment factors.

FIH hydroxylates the C-terminal activation domain (CTAD) of HIF by means of one of the oxygen atoms from O_2_, the other being transferred to the cosubstrate α-ketoglutarate (αKG), closely resembling the reaction catalyzed by PDH2. A similar solvent isotope effect study was conducted on FIH, revealing much larger inverse SKIEs on *k*_cat_ and *k*_cat_/*K*_m_ [30]. The magnitude of these inverse effects was elegantly correlated to the number of displaced Fe^2+^-bound water molecules. Assuming a fractionation factor of 0.70 for each hydron, the theoretical SIEs for one, two, and three displaced waters are 0.49, 0.24, and 0.12, respectively. The apparent ^D2O^*k*_cat_ of 0.51 ± 0.07 corresponds to a single water displaced after the second substrate, CTAD, binds to assemble the ternary complex. Because the kinetic mechanism is ordered, with CTAD binding after αKG, the apparent ^D2O^(*k*_cat_/*K*_CTAD_) of 0.40 ± 0.07 also reflects displacement of the same Fe^2+^-bound water. The first substrate, αKG, displaces two water molecules as it chelates to Fe^2+^. When CTAD is present at high concentrations, its binding becomes the first irreversible step with respect to *k*_cat_/*K*_αKG_, meaning that this parameter is insensitive to the displacement of the third water by CTAD. The apparent ^D2O^(*k*_cat_/*K*_m_)_αKG_ of 0.32 ± 0.08 is consistent with this situation. At low CTAD concentrations, however, release of the third water affects *k*_cat_/*K*_αKG_, and this is evident by the lower ^D2O^(*k*_cat_/*K*_m_)_αKG_ of 0.11 ± 0.03.

### 4.6. Adenosine and AMP Deaminases

The closely related enzymes adenosine deaminase and AMP deaminase provide an example of how inverse SIEs can be misinterpreted when information is lacking. These enzymes catalyze the hydrolytic C-6 deamination of adenosine to inosine or AMP to IMP, respectively. Weiss et al. measured ^D2O^(*k*_cat_/*K*_Ado_) = 0.77 ± 0.06 for adenosine and ^D2O^(*k*_cat_/*K*_DHOA_) = 0.45 ± 0.04 for the alternative substrate 7,8-dihydro-8-oxoadenosine (DHOA) [18]. The authors concluded that these inverse SKIEs implicate a cysteine thiol (*ϕ* = 0.4–0.5) as a Brønsted acid that is deprotonated by N1 (*ϕ*_RNL3+_ ≈ 1) of the purine during attack by water at C-6, followed by rate-limiting collapse of the tetrahedral intermediate to release ammonia. The difference in the values for the two substrates was attributed to adenosine’s 14-fold higher forward commitment to catalysis associated with release of ammonia, a factor that masks the preceding SEIE. Five years later, after the X-ray crystal structure revealed the absence of cysteine residues in the active site of adenosine deaminase [33], Cleland instead claimed that the inverse SIEs were attributable to the existence of a low-barrier hydrogen bond (LBHB; the relationship to inverse SIEs is discussed in the next section) between Glu217 and N1 of the purine having an inverse fractionation factor of ~0.4 [34].

**Figure 3:**
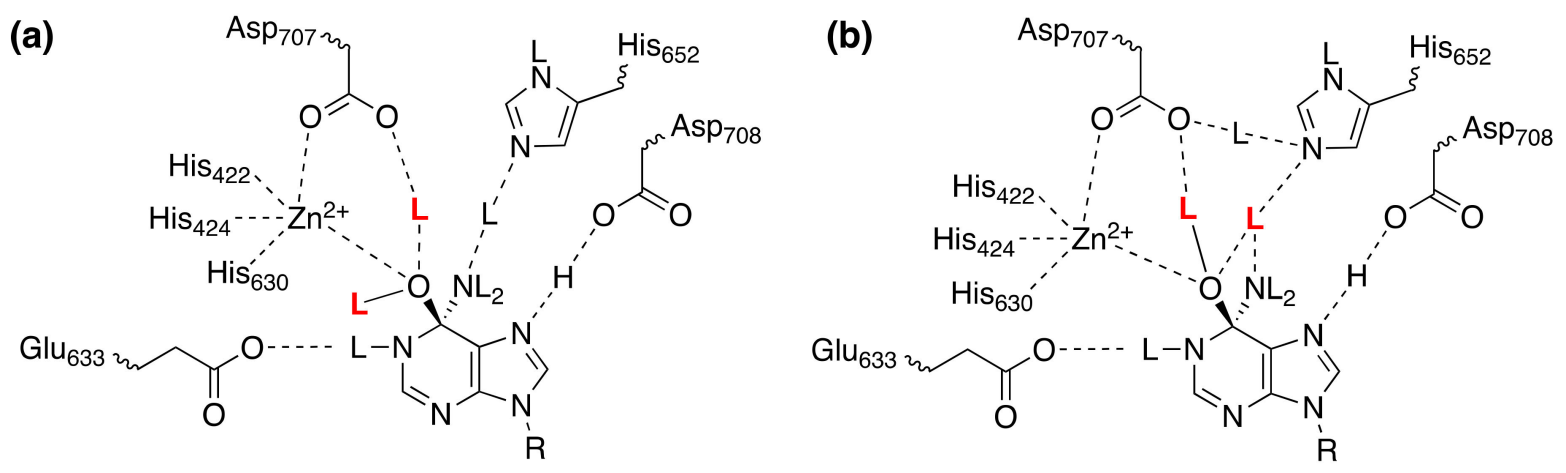
Proposed hydrogen bond network for AMP deaminase at the transition state. (**a**) Model depicting one hydron “in flight” linked to the Zn^2+^-bound water (from water to Asp707), applicable to AMP as substrate. (**b**) Model depicting two hydrons “in flight” linked to the Zn^2+^-bound water (from water to the leaving ammonia and from His652 to Asp707), applicable to 2′dAMP and 8-bromo-AMP as substrates. Figure was adapted from Merkler and Schramm [31].

The following year, Merkler and Schramm studied AMP deaminase and similarly reported inverse SKIEs on *k*_cat_/*K*_m_ for AMP and the alternative substrates 2′-dAMP and 8-bromo-AMP in the presence of 0.1 mM ATP: ^D2O^(*k*_cat_/*K*_m_) = 0.79 ± 0.11, 0.34 ± 0.02, and 0.33 ± 0.03, respectively [31]. They noted, however, that a LBHB is insufficient to explain the largely inverse effects exhibited by the alternative substrates. A single proton transfer from a Zn^2+^-bound water (^D2O^*K*_eq_ ≈ 0.75; Figure 3a) likewise falls short. A proton inventory revealed that these SKIEs involve at least two proton transfers, leading the authors to favor a proton relay involving the transfer of two protons, mediated by Asp707, His652, and the departing NH_3_ (Figure 3b). In light of this study, it is tempting to conclude that the inverse SIEs measured for both adenosine and AMP deaminases share a common origin—relief of the inverse fractionation factor of the Zn^2+^-bound nucleophilic water.

## 5. Low-Barrier Hydrogen Bonds

LBHBs occur when a proton is sandwiched at a short distance between functional groups of similar p*K*_a_ values. Because the energy barrier for proton transfer lies at or below the zero-point energy of the bond, the proton can freely associate between the flanking acceptors [35]. Although the LBHB is strong overall, the bond between the proton and either of its acceptor atoms is weak, yielding inverse fractionation factors (*ϕ*_LBHB_ = 0.30–0.60) [36].

The transfer of a proton involved in a LBHB can result in an inverse SIE under one of two conditions. First, it can be the result of a rapid-equilibrium SEIE in which the fractionation factor of the proton acceptor in the product is close to unity: ^D2O^*K*_eq_ = *ϕ*_LBHB_/*ϕ*_P_ ≈ 0.30–0.60. Alternatively, it may be the result of an intrinsic KIE for a rate-limiting proton transfer, as follows. Proton-transfer reactions originating from canonical hydrogen bonds always give rise to normal intrinsic SKIEs because the proton in-flight at the transition state has a lower fractionation factor than in the reactant: ^D2O^*k* = *ϕ*_R_/*ϕ*_TS_ > 1. However, the fractionation factor of a proton transferred from a LBHB is more inverse than at the transition state, resulting in an inverse intrinsic SKIE: ^D2O^*k* = *ϕ*_LBHB_/*ϕ*_TS_ < 1. Examples of SKIEs originating from LBHBs are provided in Table 4.

### 5.1. Aspartic Proteases

**Scheme 4:**
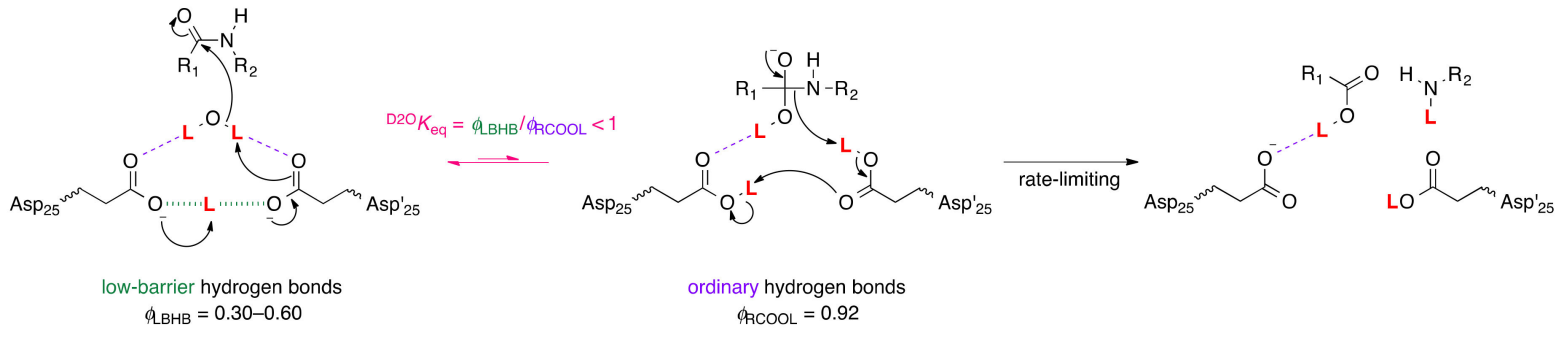
LBHBs implicated in peptide hydrolysis by aspartic proteases.

Aspartic proteases are a family of enzymes that typically utilize two aspartate residues to activate a water molecule for hydrolysis of proteins and peptides. While one oxygen from each carboxyl group forms a hydrogen bond with the catalytic water, the other oxygens share a proton in a LBHB (Scheme 4). An equilibrium for the synchronous intermolecular proton transfer in this hydrogen bond network moves a hydroxide to the amide carbon at the expense of disrupting the LBHB. The potentially rate-limiting steps following this equilibrium include collapse of the tetrahedral intermediate and slow product release. Examples of inverse ^D2O^(*k*_cat_/*K*_m_) included porcine pepsin (0.84 ± 0.21) [37] and HIV-1 protease (0.85 ± 0.09) [38].

### 5.2. Alcohol Dehydrogenase

In Section 4.3, we described how the inverse SKIEs measured for YADH and LADH could be accounted for by relief of the inverse fractionation factor of metal-bound water. A follow-up study using pre-steady-state kinetics in the LADH-catalyzed oxidation of ethanol revealed an inverse SKIE on the transient formation of NADH: ^D2O^*k*_obs_ = 0.50 [40]. A curved proton inventory for this transient was consistent with a transition-state fractionation factor (*ϕ*_TS_ = 0.73) that is less inverse than the ground-state fractionation factor (*ϕ*_R_ = 0.37). Although the concept of LBHBs had not yet been popularized at the time of this study, the authors pointed to a hydrogen bond between the alkoxide intermediate, formed prior to hydride transfer, and the alcohol of Ser48, which in turn forms a hydrogen bond network to the 2′-OH of NAD^+^ and His51. The authors argued that in the non-aqueous confines of the active site, a *ϕ* of 0.37, typical of a LBHB, could be achieved for this proton. In the transition state, this LBHB is relaxed to a typical hydrogen bond as Ser-48 associates with the product aldehyde [46]. This analysis demonstrates how an inverse SKIE can stem not from a preceding SEIE but from an intrinsic KIE in a partially rate-limiting step.

## 6. Medium Effects

Placing the substrate and enzyme in D_2_O converts all exchangeable protons to deuterons. In cases where specific hydrons cannot be assigned as the source of a SIE, a bulk medium effect resulting from the collective effect of these exchangeable sites is often invoked [1].

### 6.1. β-Lactamase

β-lactamase catalyzes the hydrolysis of acyclic depsipeptides and β-lactam antibiotics, which include penicillins, cephalosporins, and carbapenems. The mechanism of class A and class C β-lactamase-catalyzed hydrolysis of the depsipeptide *m*-((*N*-(phenylacetyl)glycyl)oxy)benzoate was investigated with β-secondary and solvent KIEs [44]. ^D2O^(*k*_cat_/*K*_m_) for the class A β-lactamase TEM (0.61–0.72) and class C β-lactamase P99 (0.79–0.85) were distinctly inverse (Table 4). The inverse nature of these SKIEs is inconsistent with general base catalysis but could be attributed to medium effects, where the fractionation factors of waters of solvation in the enzyme-bound transition state increase relative to the free enzyme and substrate, suggesting a restriction of hydron-coupled motions.

### 6.2. Alkanesulfonate Monoxygenase

Alkane monooxygenase is expressed during sulfur deprivation. It is used to tap into alternative sulfur sources by catalyzing the desulfonation of alkanesulfonates. In their mechanistic investigation of this enzyme, Robbins and Ellis measured pL–*k*_cat_ profiles at several different atom fractions of deuterium in H_2_O/D_2_O mixtures in order to construct a proton inventory [45]. The resulting unusual dome-shaped pattern was best fit by an equation that included a normal transition state effect (*ϕ*_TS_ = 0.53 ± 0.03) that was offset by a large inverse medium effect. No SKIE was observed on *k*_cat_/*K*_m_, indicating that the solvent-sensitive steps occur after the first irreversible step. The *ϕ*_TS_ contribution to the SKIE was attributed to proton transfer from the catalytic base, Arg226, to the oxygen of the anionic flavin intermediate. The medium effect, on the other hand, was attributed to a conformational change that occurs to open up the enzyme, allowing release of the product.

### 6.3. Glucokinase

**Scheme 5:**
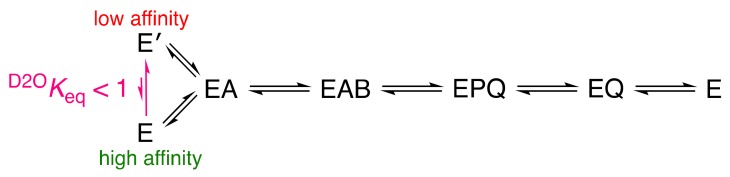
So-called mnemonical mechanism for glucokinase proposed by Storer and Cornish-Bowden [47]. E and E′ are high- and low-affinity conformations, respectively, of glucokinase. A = glucose; B = ATP; P = ADP; Q = glucose 6-phosphate.

Glucokinase is the primary hexokinase isozyme that phosphorylates *O*-6 of glucose. In H_2_O, it exhibits positive cooperativity with respect to glucose. Storer and Cornish-Bowden accounted for this behavior by invoking two forms of the free enzyme, E and E′, to which glucose binds with high and low affinities, respectively (Scheme 5) [47]. At low concentrations of glucose, E is allowed to relax to E′ after every turnover, whereas, when levels are high, glucose is able to intercept E prior to relaxation to E′. In D_2_O, the cooperativity becomes negative and exhibits an exceptionally large inverse SKIE of 0.22 at low glucose concentration (0.1 mM); the SKIE increases with increasing glucose, eventually becoming normal (1.3) at saturation [42].

Two explanations were offered for the inverse SKIE. First, D_2_O stabilizes E, such that its tendency to relax to E′ is diminished. As the enzyme becomes saturated, however, the involvement of E and E′ is diminished, revealing a small, normal SKIE from another step in the mechanism. Alternatively, D_2_O causes an increase in glucose affinity of E′, such that the reaction is accelerated in D_2_O relative to H_2_O. Unfortunately, no definitive mechanism could be proposed to account for either of these possibilities, although a medium effect was suggested as a contributor. What is clear, however, is that the inverse SIE is an equilibrium effect.

## 7. Protein Conformational Changes

Inverse SKIEs are sometimes observed in systems with no obvious origin but where a mechanistically relevant conformational change is involved. As the following examples illustrate, the exact nature of the isotope perturbation is unknown, but could involve changes in hydrogen bonding, including medium effects like those described in the previous section.

### 7.1. β-Lactam Synthetase

β-lactam synthetase forms the β-lactam core in the biosynthesis of clavulanic acid, an inhibitor of β-lactamase, which deactivates β-lactam antibiotics through hydrolysis. An inverse SKIE of 0.67 ± 0.15 on *k*_cat_/*K*_m_ was measured for the cyclization of *N*^2^-(2-carboxyethyl)-L-arginine to deoxygaunidinoproclavaminic acid by β-lactam synthetase [39]. Due to the lack of a cysteine residue in the active site and of any germane substrate association to a metal cofactor, this inverse SKIE was attributed to torsional strain as the enzyme switches from an open to a closed conformation.

### 7.2. β-Ketoacyl Carrier Protein Reductase

β-ketoacyl carrier protein (ACP) reductase is part of the type-II fatty acid elongation system in bacterial fatty acid biosynthesis. It is a potential drug target, as mammals use the type-I pathway for the same process. In order to delineate the mechanism of the enzyme from *M. tuberculosis*, MabA, solvent and NADPD KIEs were measured, along with mixed isotope effects, as follows [41]. First, a primary deuterium KIE of 2.5 ± 0.1 was measured on *k*_cat_/*K*_NADPH_ (i.e., with NADPH as the varied substrate), indicating that hydride transfer is partially rate limiting. Next, a small SKIE of 1.30 ± 0.02 was measured on the same parameter using NADPH as the varied substrate, indicating that at least one proton-transfer step is partially rate limiting on the second-order rate constant. In order to assess the concerted/stepwise nature of the hydride and proton transfers, a mixed KIE was determined by measuring the SKIE in the presence of NADPD: ^D2O^(*k*_cat_/*K*_NADPD_) = 0.58 ± 0.04. This strikingly inverse SKIE supports a stepwise mechanism since, in a concerted mechanism, NADPD would make the common step more rate limiting, increasing ^D2O^(*k*_cat_/*K*_NADPH_). The authors suggested that the inverse SKIE may be due to an inverse SEIE that precedes rate-limiting chemistry, a pattern that has been seen throughout this review. Analogy was drawn to 2-*trans*-enoyl-ACP reductase from *M. tuberculosis*, which exhibited an inverse SKIE on *k*_cat_/*K*_m_ using dodecanoyl-CoA as the varied substrate [48]; the effect was attributed to a conformational change during or following substrate binding, though the exact isotope perturbation was not addressed. MabA likewise undergoes a conformational change when NADPH binds [49], so a similar effect may also be at play with this enzyme.

## 8. Solvent Viscosity Effects vs. Solvent Isotope Effects

When performing SIE studies, it is imperative to carry out the reaction in both light and heavy water. D_2_O is 24% more viscous than H_2_O, and the change in viscosity alone may be responsible for observed isotope effects. It is therefore prudent to check the kinetic parameters in question under conditions of comparable viscosity using viscosogens to delineate the contributions from isotope-sensitive steps and diffusion-limited steps.

### NAD-Malic Enzyme

NAD-malic enzyme utilizes NAD^+^ to oxidatively decarboxylate malate, yielding pyruvate, CO_2_, and NADH. The reaction involves hydride transfer from C-2 of malate and deprotonation of its 2-hydroxyl group. In an attempt to determine whether these two processes are stepwise or concerted, Karsten et al. performed a SKIE investigation using various fixed and varied substrates and cofactors [50]. As can be seen in Table 5, nearly all SKIEs were inverse, yet the common sources of inverse SIEs (i.e., thiol groups, metal-bound water, and medium effects) were excluded for this enzyme. The authors wisely measured solvent viscosity effects by matching the viscosity of D_2_O using glycerol as a microviscosogen (Table 5). Strikingly, the SKIEs generally matched the solvent viscosity effects. Increasing the viscosity slows down diffusion-limited steps such as substrate binding or product release, but these generate viscosity effects in the opposite direction (i.e., slower in solvents with higher viscosity) from those observed with NAD-malic enzyme. The authors suggested instead that the viscosity effects were most likely related to changes in enzyme conformation (or rates of such changes) during catalysis. This study demonstrated a crucial lesson: all solvent isotope effect studies should employ viscosity controls, preferably with at least two different viscosogens, since they may manifest different viscosity effects [50].

## 9. Conclusions

Inverse SKIEs are uncommon but, when observed, may be indicative of a SEIE. To determine the origin of the inverse SKIE, the enzyme should first be inspected for catalytically relevant cysteines or metal-bound waters and, if present, a theoretical SEIE should be calculated from known fractionation factors. Although rare, the existence of a LBHB can be considered if it is supported by other experimental data such as structure and matched donor and acceptor p*K*_a_ values. In the absence of the above features, the inverse SKIE may be broadly explained by invoking medium effects, where ground state vibrational motions are restricted in a rapid equilibrium prior to rate-limiting steps or restricted at the rate-limiting transition state. Minimally, inverse SIEs can be used to exclude proton-transfer steps as being rate limiting, which may bring clarity to enzyme mechanisms.

## Figures and Tables

**Table 1:** Common fractionation factors implicated in enzyme-catalyzed reactions.

Exchangeable Group	*ϕ*	Reference
L_3_O^+^	0.69	[3]
OL^−^	0.43	[4]
RSL (Cys)	0.55	[5]
ROL (Ser)	1.04	[6]
RCO_2_L (Asp, Glu)	0.92	[6]
RNL_2_ (His, Lys, Arg)	1.13	[6]
RNL_3_^+^ (His, Lys, Arg)	1.08	[7]
M–OL (M = Co^2+^)	0.77	[8]
M–OL_2_ (M = Co^2+^)	0.81	[8]

**Table 2:** Examples of inverse solvent isotope effects due to cysteine thiols.

Enzyme	Varied Substrate/Agent	Inverse SKIE ^1^	Reference
papain	Bz-Gly-OPh	^D2O^*k*_ac_ = 0.63	[14]
	Bz-Arg-NH_2_	^D2O^*k*_ac_ = 0.58	[14]
	Bz-Cit-NH_2_	^D2O^*k*_ac_ = 0.46	[14]
	Z-Phe-Gly-NH_2_	^D2O^*k*_ac_ = 0.63	[14]
	bromoacetamide	^D2O^*k*_alk_ = 0.74	[14]
cysteine (papain model)	bromoacetate	^D2O^*k*_alk_ = 0.70 ± 0.02	[15]
	chloroacetate	^D2O^*k*_alk_ = 0.85 ± 0.10	[15]
	bromoacetamide	^D2O^*k*_alk_ = 0.69 ± 0.10	[15]
	chloroacetamide	^D2O^*k*_alk_ = 0.64 ± 0.04	[15]
	methyl bromoacetate	^D2O^*k*_alk_ = 0.53 ± 0.03	[15]
cathepsin C	Ser-Tyr-AMC	^D2O^(*k*_cat_/*K*_m_) = 0.71 ± 0.14	[9]
protein arginine deiminase	Bz-Arg-OEt (PAD2)	^D2O^(*k*_cat_/*K*_m_) = 0.8 ± 0.1	[16]
	Bz-Arg-OEt (PAD4)	^D2O^(*k*_cat_/*K*_m_) = 0.43	[17]
isocitrate lyase	succinate	^D2O^(*k*_cat_/*K*_m_) = 0.56 ± 0.07	[10]
adenosine deaminase	adenosine 7,8-dihydro-8-oxoadenosine	^D2O^(*k*_cat_/*K*_m_) = 0.77 ± 0.06	[18]
		^D2O^(*k*_cat_/*K*_m_) = 0.45 ± 0.04	[18]

^1^*k*_ac_ = the rate constant for enzyme acylation; *k*_alk_ = the rate constant for enzyme alkylation.

**Table 3:** Examples of inverse solvent isotope effects due to metal-bound water.

Enzyme Metal Cofactor	Varied Substrate/Condition	Inverse SKIE	Ref.
carbonic anhydrase C (esterase activity); Zn^2+^	*p*-nitrophenyl acetate	^D2O^(*k*_cat_/*K*_m_) = 0.73	[22]
thermolysin; Zn^2+^	3-(2-furyl)acrolyl-Gly-Leu-NH_2_ 3-(2-furyl)acrolyl-Gly-Ala-NH_2_ 3-(2-furyl)acrolyl-Gly-Abu-NH_2_ 3-(2-furyl)acrolyl-Gly-Val-NH_2_ 3-(2-furyl)acrolyl-Gly-Nva-NH_2_ 3-(2-furyl)acrolyl-Gly-Phe-NH_2_ 3-(2-furyl)acrolyl-Gly-Leu-NH_2_ 3-(2-furyl)acrolyl-Gly-Leu-Ala	^D2O^(*k*_cat_/*K*_m_) = 0.80 ± 0.02 ^D2O^(*k*_cat_/*K*_m_) = 0.80 ± 0.01 ^D2O^(*k*_cat_/*K*_m_) = 0.73 ± 0.01 ^D2O^(*k*_cat_/*K*_m_) = 0.76 ± 0.01 ^D2O^(*k*_cat_/*K*_m_) = 0.73 ± 0.01 ^D2O^(*k*_cat_/*K*_m_) = 0.73 ± 0.02 ^D2O^(*k*_cat_/*K*_m_) = 0.69 ± 0.02 ^D2O^(*k*_cat_/*K*_m_) = 0.72 ± 0.01	[23] [24] [24] [24] [24] [24] [24] [24]
stromelysin; Zn^2+^	pL 6.0 pL 7.5 pL 9.5	^D2O^(*k*_cat_/*K*_m_) = 0.80 ± 0.03 ^D2O^(*k*_cat_/*K*_m_) = 0.85 ± 0.06 ^D2O^(*k*_cat_/*K*_m_) = 0.75 ± 0.02	[25] [25] [25]
desuccinylase; Zn^2+^	*N*-succinyl-L,L-diaminopimelate	^D2O^(*k*_cat_/*K*_m_) = 0.62 ± 0.06	[26]
		^D2O^*k*_cat_ = 0.78 ± 0.05	[26]
alcohol dehydrogenase; Zn^2+^	NADH NADD	^D2O^*k*_cat_ = 0.50 ± 0.05 ^D2O^*k*_cat_ = 0.58 ± 0.06	[27] [27]
	pL 8.75, single turnover	^D2O^*k*_slow_ = 0.394 ± 0.005	[28]
malate synthase; Mg^2+^	[*methyl*-^2^H_3_]acetyl-CoA	^D2O^(*k*_cat_/*K*_m_) = 0.6 ± 0.1	[29]
prolyl hydroxylase 2; Fe^2+^	acidic basic	^D2O^*k*_cat_ = 0.91 ± 0.03 ^D2O^*k*_cat_ = 0.9 ± 0.1	[11] [11]
factor-inhibiting hypoxia-inducible factor; Fe^2+^	saturating CTAD, αKG CTAD αKG (high [CTAD]) αKG (low [CTAD])	^D2O^*k*_cat_ = 0.51 ± 0.07 ^D2O^(*k*_cat_/*K*_m_) = 0.40 ± 0.07 ^D2O^(*k*_cat_/*K*_m_) = 0.32 ± 0.08 ^D2O^(*k*_cat_/*K*_m_) = 0.11 ± 0.03	[30] [30] [30] [30]
adenosine deaminase; Zn^2+^	adenosine 7,8-dihydro-8-oxoadenosine	^D2O^(*k*_cat_/*K*_m_) = 0.77 ± 0.06 ^D2O^(*k*_cat_/*K*_m_) = 0.45 ± 0.04	[18] [18]
AMP deaminase; Zn^2+^	AMP, no ATP	^D2O^(*k*_cat_/*K*_m_) = 0.71 ± 0.07	[31]
		^D2O^*k*_cat_ = 0.98 ± 0.04	[31]
	AMP, 0.1 mM ATP	^D2O^(*k*_cat_/*K*_m_) = 0.79 ± 0.11	[31]
		^D2O^*k*_cat_ = 0.75 ± 0.03	[31]
	2′-dAMP, no ATP	^D2O^(*k*_cat_/*K*_m_) = 0.49 ± 0.04	[31]
		^D2O^*k*_cat_ = 0.62 ± 0.02	[31]
	2′-dAMP, 0.1 mM ATP	^D2O^(*k*_cat_/*K*_m_) = 0.34 ± 0.02	[31]
		^D2O^*k*_cat_ = 0.63 ± 0.02	[31]
	8-Br-AMP, 0.1 mM ATP	^D2O^(*k*_cat_/*K*_m_) = 0.33 ± 0.03	[31]
		^D2O^*k*_cat_ = 0.58 ± 0.03	[31]

**Table 4:** Examples of inverse solvent isotope effects (SIEs) from low-barrier hydrogen bonds (LBHBs) and medium effects.

Enzyme	Substrate	Inverse SKIE	Ref.
pepsin	Leu-Ser-(*p*-NO_2_)-Phe-Nle-Ala-Leu-OMe	^D2O^(*k*_cat_/*K*_m_) = 0.84 ± 0.21	[37]
HIV-1 protease		^D2O^(*k*_cat_/*K*_m_) = 0.85 ± 0.09	[38]
β-lactam synthetase	*N*^2^-(2-carboxyethyl)-l-arginine	^D2O^(*k*_cat_/*K*_m_) = 0.67 ± 0.15	[39]
alcohol dehydrogenase	ethanol, saturating NAD^+^	^D2O^(*k*_obs_) = 0.50 ^1^	[40]
ACP reductase	AcAcSCoA, NADPD	^D2O^(*k*_cat_/*K*_m_) = 0.58	[41]
glucokinase	glucose, low ATP glucose, high ATP	^D2O^(*k*_cat_/*K*_m_) = 0.22 ^D2O^(*k*_cat_/*K*_m_) = 0.29	[42] [42]
fructose 1,6-bisphosphate aldolase	WT E168A D276A	^D2O^(*k*_cat_/*K*_m_) = 0.94 ± 0.07 ^D2O^(*k*_cat_/*K*_m_) = 0.61 ± 0.07 ^D2O^(*k*_cat_/*K*_m_) = 0.79 ± 0.06	[43] [43] [43]
TEM (class A β-lactamase)	depsipeptide, ^2^ pL 7.0 depsipeptide, ^2^ pL 7.5 depsipeptide, ^2^ pL 8.0	^D2O^(*k*_cat_/*K*_m_) = 0.72 ± 0.03 ^D2O^(*k*_cat_/*K*_m_) = 0.70 ± 0.02 ^D2O^(*k*_cat_/*K*_m_) = 0.61 ± 0.02	[44] [44] [44]
P99 (class C, β-lactamase)	depsipeptide, ^2^ pL 7.5	^D2O^(*k*_cat_/*K*_m_) = 0.79 ± 0.01 ^3^ ^D2O^(*k*_cat_/*K*_m_) = 0.85 ± 0.01 ^3^	[44] [44]
alkanesulfate monooxygenase	1-octanesulfonate	^D2O^(*k*_cat_/*K*_m_) = 0.95 ± 0.07	[45]
		^D2O^(*k*_cat_) = 0.75 ± 0.04	[45]
	1-[1,1-^2^H_2_]-octanesulfonate	^D2O^(*k*_cat_/*K*_m_) = 0.83 ± 0.17	[45]
		^D2O^(*k*_cat_) = 0.79 ± 0.07	[45]

^1^*k*_obs_ is the observed rate constant under pre-steady-state conditions. ^2^ Depsipeptide = *m*-[[*N*-(phenylacetyl)glycyl]oxy]benzoate. ^3^ Independent experiments.

**Table 5:** Inverse solvent isotope and solvent viscosity effects for NAD-malic enzyme ^1^.

Substrate	Fixed Substrate, Cofactor(s)	SKIE		Solvent Viscosity Effect ^2^	
		^D2O^(*k*_cat_/*K*_m_)	^D2O^ *k*_cat_	*k*_cat_/*K*_m_	*k*_cat_
malate	NAD^+^, Mg^2+^	0.5 ± 0.1	0.76 ± 0.02	0.6 ± 0.2	0.85 ± 0.07
	NAD^+^, Cd^2+^	0.37 ± 0.06	1.0 ± 0.2	0.61 ± 0.02	0.86 ± 0.08
	NAD^+^, Mn^2+^	0.5 ± 0.3	1.272 ± 0.006	0.62 ± 0.05	1.0 ± 0.1
	NAD^+^, Mg^2+^, fumarate	0.47 ± 0.07	0.80 ± 0.02	0.46 ± 0.03	0.84 ± 0.01
	APAD, ^3^ Mg^2+^	0.49 ± 0.06	0.91 ± 0.02	0.62 ± 0.06	0.82 ± 0.06
	APAD, Mn^2+^	0.76 ± 0.03	0.99 ± 0.01	0.57 ± 0.01	0.78 ± 0.04
	APAD, Mn^2+^, fumarate	0.81 ± 0.03	1.07 ± 0.02	0.68 ± 0.02	0.85 ± 0.03
NAD^+^	malate, Mg^2+^	0.57 ± 0.02	0.81 ± 0.06	0.689 ± 0.001	0.891 ± 0.001
pyruvate	NADH, CO_2_, Mg^2+^	0.42 ± 0.01	0.35 ± 0.08	0.41 ± 0.02	0.44 ± 0.07

^1^ Data taken from Karsten et al. [50]. ^2^ Relative solvent viscosity using 9% glycerol as a viscosogen, expressed as the ratio of rate constants, (*η*_rel_ = 1)/(*η*_rel_ = 1.24). ^3^ APAD = 3-acetylpryidine adenine dinucleotide.
